# Assessment of the maternal key micronutrient supply and its correlation with cord blood parameters in twin pregnancies

**DOI:** 10.3389/fpubh.2025.1487730

**Published:** 2025-02-25

**Authors:** Magdalena Zgliczynska, Magdalena Ostrowska, Kinga Zebrowska, Iga Rzucidlo-Szymanska, Iwona Szymusik, Konrad Kowalski, Katarzyna Kosinska-Kaczynska

**Affiliations:** ^1^Department of Obstetrics, Perinatology and Neonatology, Centre of Postgraduate Medical Education, Warsaw, Poland; ^2^Doctoral School of Translational Medicine, Centre of Postgraduate Medical Education, Warsaw, Poland; ^3^Department of Endocrinology, Centre of Postgraduate Medical Education, Warsaw, Poland; ^4^Masdiag Sp. z o.o., Warsaw, Poland

**Keywords:** twin pregnancy, cord blood, micronutrient, iron, vitamin B12, folic acid, vitamin D

## Abstract

**Background:**

Multiple pregnancy constitutes a large metabolic expense, so women with twin pregnancies and neonates born as twins might be at risk for micronutrient deficiencies. Therefore, the aim of the study was to assess the supplementation used and supply with key micronutrients: iron, vitamin B12, folic acid and vitamin D in women with twin pregnancies and the correlations with cord blood indicators.

**Methods:**

Maternal and cord blood samples were collected from 51 patients with twin pregnancies and 102 newborns born from those pregnancies between October 2020 and September 2023. Ferritin, vitamin B12, folic acid and vitamin D metabolites concentrations were measured. Additionally, the patients completed a questionnaire regarding pre-and intragestational supplementation.

**Results:**

Iron, vitamin B12, and vitamin D deficiency were diagnosed in 20.8, 13.7 and 12.5% of women with twin pregnancies, respectively. No maternal folate deficiency was identified. Positive weak to moderate correlations were demonstrated between the concentrations of all studied indicators in the maternal and cord blood. Pregestational folic acid and vitamin D supplementation was associated with higher cord blood levels of folic acid and 3-epi-25(OH)D3, respectively. 25(OH)D3 and total 25(OH)D concentrations were higher in newborns whose mothers had supplemented vitamin D during pregnancy.

**Conclusion:**

The problem of iron, vitamin B12 and vitamin D deficiencies in twin pregnancies is still valid. Pre-and intragestational supplementation, as well as maternal micronutrient supply affect the cord blood composition of twins.

## 1 Introduction

In recent decades, the incidence of twin pregnancies has significantly increased (1). According to an analysis published by Monden et al. in 2021, an increase in twinning rate of over 10% was noted in 74 out of 112 studied countries between the periods of 1980–1985 and 2010–2015 (1). This phenomenon is primarily due to the spread of assisted reproductive technologies. Other known factors might also contribute to increasing the chances of conceiving twins: advanced maternal age or the increasing preconception body mass index in some regions of the world (2, 3).

Multiple pregnancy constitutes a large metabolic expense, greater than a physiological singleton pregnancy (4, 5). This may lead to an increased risk of nutritional deficiencies. Available studies showed that women with multiple pregnancies might be at a higher risk of vitamin D and iron deficiencies, whereas for other microelements, the data are scarce or absent (6–9). However, two available studies showed no increased risk of folic acid and vitamin B12 deficiencies at the time of delivery in women with multiple pregnancies (10, 11). Nevertheless, to our knowledge, no official guidelines were issued by scientific societies regarding supplementation and nutrition in twin gestation. Considering that these are pregnancies associated with a higher risk of both maternal and fetal complications, it seems particularly important to influence the modifiable risk factors, such as nutritional deficiencies and related secondary diseases, such as anemia (12, 13).

The placenta is an organ that, apart from a number of other important functions, enables the transplacental transport of nutrients from the maternal body to the growing fetus or fetuses (14). Consequently, they are dependent on maternal nutrient stores (15–17). Multiple pregnancy might constitute a risk factor for neonatal vitamin D deficiency as well as neonatal deficiency anemia. Therefore, fetuses in multiple pregnancies might receive fewer or, perhaps, too few, nutrients during pregnancy (8, 18).

Therefore, the main aim of the study involved the assessment of the concentration of key micronutrients: iron, folic acid, vitamin B12 and vitamin D in pregnant women with twin pregnancies and the respective correlations with cord blood indicators. An additional goal was to evaluate supplementation and its impact on maternal and cord blood parameters in this cohort.

## 2 Materials and methods

### 2.1 General information

The STROBE (Strengthening the Reporting of Observational studies in Epidemiology) Guidelines were followed for reporting the study (19). This study is part of a larger project aimed at analyzing selected indicators of the nutritional status of pregnant women with twin pregnancies in terms of key vitamins and trace elements. Data on the vitamin D status in singleton and twin pregnancies were previously published (20). The final study group included 51 patients with twin pregnancies and 102 newborns born from those pregnancies. The recruitment was carried out from October 2020 to September 2023 among patients with twin pregnancies hospitalized at a tertiary center, i.e., Department of Obstetrics, Perinatology and Neonatology, Center of Postgraduate Medical Education in Warsaw. A total of 27.4% of samples were collected during calendar winter, 19.6, 25.5 and 27.5% during spring, summer and autumn, respectively (*p* > 0.05).

Appropriate approval was obtained from the local ethics committee at the Center of Postgraduate Medical Education (reference number 46/PB/2020 and 85/PB/2020), and all included patients gave their informed and written consent. All the women received an original survey containing demographic questions as well as data on the supplementation and dosage of folic acid and vitamin D used before pregnancy (at least for 1 month before obtaining a positive pregnancy test) and the supplementation and dosage of iron, vitamin B12, folic acid, and vitamin D during pregnancy (at least for the last month before birth) along with the brand name if a multiple supplement was used. We assessed the concentrations of ferritin, folic acid, vitamin B12, and vitamin D metabolite panel (25-hydroxyvitamin D2 [25(OH)D2], 25-hydroxyvitamin D3 [25(OH)D3], 3-epi-25-hydroxyvitamin D3 [3-epi-25(OH)D3], 24,25-dihydroxyvitamin D [24,25(OH)2D]) in the maternal and cord blood, and the blood count and C-reactive protein (CRP) in maternal samples.

Pregnant women were counseled in the outpatient clinic. The inclusion criteria were as follows: the ability to make independent legal decisions about oneself, a live twin pregnancy above 24 + 0 weeks, gestational age calculated basing on the last menstrual period and verified by the crown–rump length in the first trimester of pregnancy. The exclusion criteria were the lack of informed consent and known disorders affecting the metabolism of the studied micronutrients (hyper- and hypoparathyroidism, rickets, sarcoidosis, thalassemia, hemochromatosis, pernicious anemia, celiac disease, inflammatory bowel diseases, any congenital defects of iron, calcium, iron, vitamin B12, folic acid or vitamin D metabolism), and the diagnosis of twin-to-twin transfusion syndrome, twin anemia-polycythemia sequence, twin reversed arterial perfusion syndrome or selective fetal growth restriction.

### 2.2 Statistical analysis

Data were presented as medians with interquartile ranges. The Mann–Whitney *U* test was used to compare two groups, and the Wilcoxon test was used if related groups were compared. Correlation analysis was performed using the Spearman’s rank correlation coefficient. The result of 0–0.19 was considered as very weak, 0.2–0.39 weak, 0.40–0.59 moderate, 0.6–0.79 strong and 0.8–1 as a very strong correlation. Analyses of more than two groups were performed using the Kruskal-Wallis test. Calculations were performed in the R language in the RStudio environment and the Statistica 13 software (TIBCO Software Inc., California, US). *p* values lower than 0.05 were considered significant. In order to assess the association between maternal and neonatal concentrations of the studied parameters, a linear regression model was used on those variables that presented a normal distribution in the graphical evaluation using a histogram and a QQ chart. Due to the presence of outliers, 2 values of 25(OH)D2 in the cord blood samples and 2 values of 25(OH)D3 in the maternal samples were removed. The R2 coefficient, which is the percentage of explained variance, and the regression coefficients for individual variables along with their standard error (SE) were used to present the model. In the case of multivariate analysis, the adjusted R2 was used, with a correction for the multivariate model.

### 2.3 Adopted definitions

A serum level of total 25(OH)D (calculated as 25(OH)D2 + 25(OH)D3) of 30–50 ng/mL was assumed to indicate an optimal supply of vitamin D (21). Ferritin <30 μg/L was considered to be diagnostic of iron deficiency (22). As regards folic acid and vitamin B12, we used the standards proposed by the laboratory, adapted to the used kits, i.e., 3.89–26.8 ng/mL and 197–771 pg/mL, respectively. Body mass index (BMI) was calculated: body weight [kilograms]/height [meters] (2). Gestational weight gain was calculated by subtracting body weight before birth and pregestational body weight.

### 2.4 Sample collection

#### 2.4.1 Collection and processing of material

Maternal samples were collected at a single time point via venipuncture, after overnight fasting into 6.0 mL Vacuette tubes with a serum clot activator within 48 h before delivery. Accordingly, cord blood samples were collected immediately after delivery. The tubes were left at room temperature for 30 min to allow clotting, then centrifuged at 2000 g for 10 min. The serum samples were aliquoted and stored at −30°C.

#### 2.4.2 The determination of 25(OH)D and other vitamin D metabolites using LC–MS/MS

The determination of vitamin D metabolites was performed in Masdiag laboratory located in Warsaw, which participates in multiple interlaboratory proficiency testing programs, including Vitamin D External Quality Assessment Scheme (DEQAS). The isotope dilution LC–MS/MS was used to analyze vitamin D metabolites (24,25(OH)2D3, 25(OH)D2, 25(OH)D3, 3-epi-25(OH)D3). The samples (20 μL) were subjected to LC–MS/MS analysis. The testing was conducted using the ExionLC analytical high-performance liquid chromatography (HPLC) system with CTC PAL (Zwinger, Switzerland) autosampler coupled to the QTRAP® 5,500 MS/MS system (Sciex, Framingham, MA, USA). All analyses were carried out in a positive mode using electrospray ionization. For quantitative analysis, multiple reaction monitoring was used. Chromatographic analyses were performed using Eclipse XDB-C18 1.7 m (50 × 4.6 mm) with a titanium prefilter at a flow rate of 0.8 mL/min. The column oven temperature was 40°C. The mobile phase included water (A) and acetonitrile (B), both with the addition of 0.1% formic acid. The total run time was 5.5 min.

#### 2.4.3 The determination of folic acid, vitamin B12 and ferritin

Folic acid, vitamin B12, and ferritin were measured in the Polish network of certified laboratories (Diagnostyka) with the use of the Cobas e801 analyzer (Roche Diagnostics, UK) and the electrochemiluminescence (ECLIA) method. Vitamin B12 and ferritin were determined using a competitive assay, while folate was measured using the sandwich assay format. The inter-assay and intra-assay coefficients of variation (CV) for folate were 4.0–7.8% and 3.6–7.3%, respectively. The respective coefficients for vitamin B12 were 1.8–8.1% and 1.6–7.2%, and for ferritin, they were 2.1–7.1% and 1.1–6.4%. The detection limit was 120 ng/mL for folate, 100 pg/mL for vitamin B12, and 0.5 ng/mL for ferritin.

## 3 Results

### 3.1 Study group characteristics

**Figure 1:**
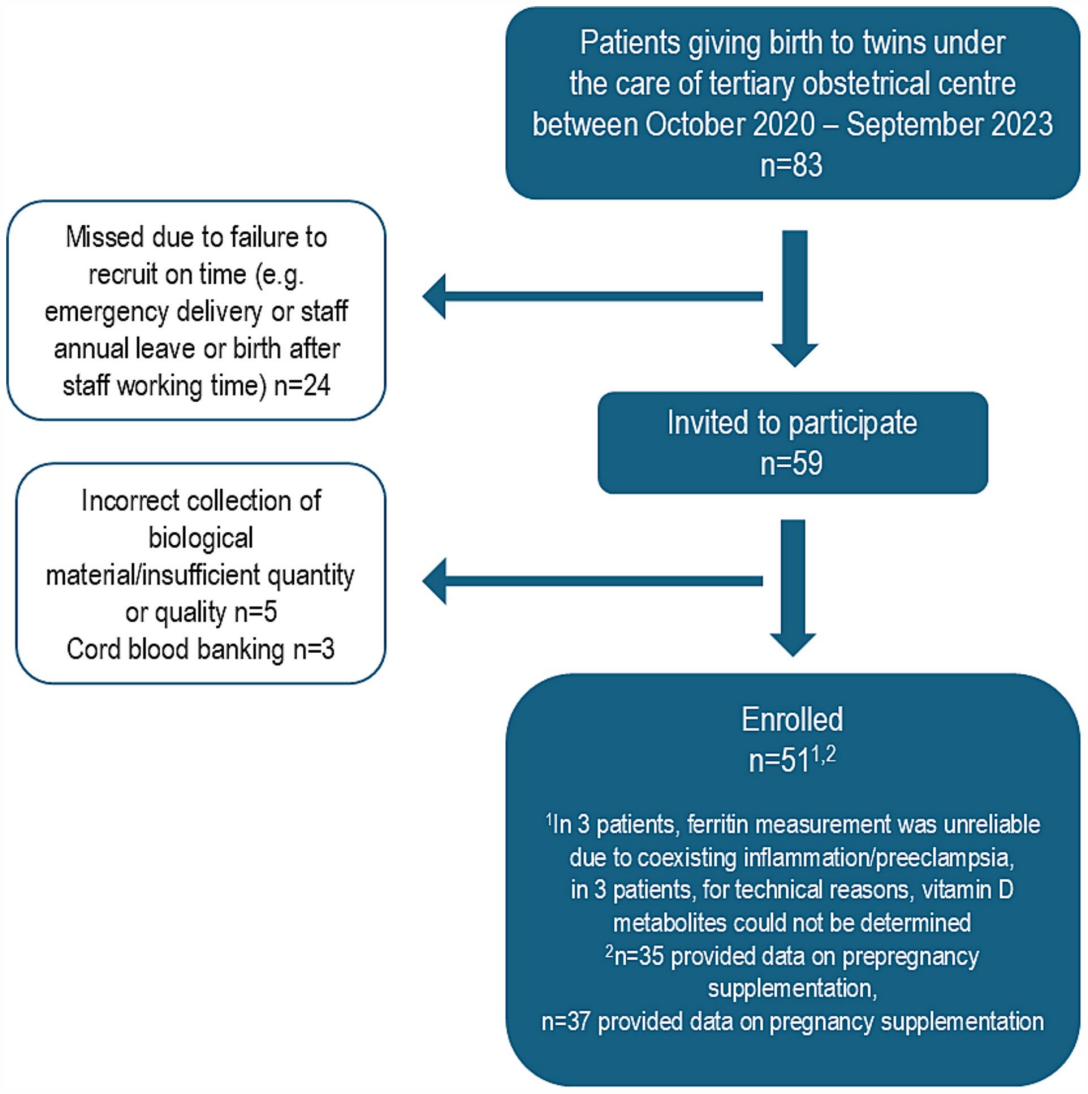
Detailed recruitment process.

A detailed description of the recruitment process is presented in Figure 1.

The characteristics of the study group are presented in Table 1. Monochorionic pregnancies constituted 37.3% (*n* = 19) of the study group. Women with monochorionic pregnancies had lower pregestational BMI and a higher incidence of gestational diabetes mellitus and preterm birth, whereas the birth weight of newborns born from those pregnancies was statistically lower (Table 1). The median hemoglobin was 11.7 g/dL. The anemia rate was 21.6%. In 8/11 cases, it was mild (Hb >10.0 g/dL) and the lowest detected hemoglobin level was equal to 8.7 g/dL. The comparison of the characteristics of patients with monochorionic and dichorionic pregnancies is provided in Supplementary material 1.

**Table 1:** Characteristics of the study group.

Characteristics	Data presentation	Results
51 women pregnant with twins		
**Maternal age [years]**	Median (IQR)	33.0 (29.0; 35.0)
**Parity**	% (*n*)	
⋅Primiparous		45.1 (23)
⋅Multiparous		54.9 (28)
**Type of twin pregnancy**	% (*n*)	
⋅Monochorionic		37.3 (19)
⋅Dichorionic		62.7 (32)
**Conception**	% (*n*)	
⋅Spontaneous		72.5 (37)
⋅ART		27.5 (14)
**Pregestational BMI [kg/m^2^]**	Median (IQR)	23.4 (21.2; 25.7)
**Pregnancy complications**	% (*n*)	
⋅GDM		9.8 (5)
⋅HDP		7.8 (4)
⋅PB		60.8 (31)
**Gestational weight gain [kg]**	Median (IQR)	17 (11; 22)
**Gestational age at birth** **[weeks+days]**	Median (IQR)	36 + 6 (35 + 1; 37 + 0)
102 neonates born from twin pregnancies		
**Birth weight [grams]**	Median (IQR)	2,435 (2050; 2,720)
Appropriate for gestational age	% (*n*)	92.2 (94)
Small for gestational age		7.8 (8)
Large for gestational age		0.0 (0)
**Initial Apgar score [points]**	% (*n*)	
8–10		94.1 (96)
4–7		5.9 (6)
0–3		0.0 (0)

ART, Assisted Reproductive Technology; BMI, Body Mass Index; GDM, Gestational Diabetes Mellitus; HDP, Hypertensive Disorders of Pregnancy; PB, Preterm Birth.

### 3.2 Declared maternal supplementation

A total of 72.5% (*n* = 37) of women completed the supplementation survey. However, 2 of them did not provide data on pregestational supplementation composition (see details in Table 2).

**Table 2:** Declared maternal supplementation.

Supplementation	Prevalence of supplementation use	Median*	1st quartile*	3rd quartile*
Folic acid (pregestational) [μg/day]	54.3%	0.4	0.4	0.4
Vitamin D (pregestational) [IU/day]	42.9%	2000.0	2000.0	2000.0
Iron [mg/day]	75.7%	30.0	27.0	107.0
Vitamin B12 [μg/day]	51.4%	4.0	3.0	4.5
Folic acid [μg/day]	100%	0.6	0.4	0.8
Vitamin D [IU/day]	94.6%	2000.0	2000.0	2000.0

IU, international units.

*the presented median and quartiles are calculated only for women who supplemented a particular micronutrient.

Taking account of the guidelines of the Polish Society of Obstetricians and Gynecologists regarding supplementation recommended during pregnancy valid at the time of the study, 27.0% of the patients did not take (*n* = 2/37) or took too low (*n* = 8/37) doses of vitamin D (<2000 IU/day) and 40.5% (*n* = 15/37) took too low doses of folic acid (<0.6 μg/day) (23). In 50% of cases of iron supplementation, the element was used unnecessarily or in excessive doses relative to the current hemoglobin and ferritin status (24). All patients supplemented vitamin D in the form of cholecalciferol.

### 3.3 Concentrations of the studied nutrients or their indicators in the maternal blood

Concentrations of ferritin, vitamin B12, folic acid and vitamin D assessed in maternal blood samples are presented in Table 3. Low serum ferritin concentrations were observed in 20.8% (*n* = 10/48) of patients which indicated iron deficiency, whereas 13.7% (*n* = 7/51) of women had their vitamin B12 concentrations below the reference ranges for the used assay. No maternal folic acid deficiency was identified. We found vitamin D deficiency in 12.5% (*n* = 6/48) of patients.

**Table 3:** Concentrations of ferritin, vitamin B12, folic acid and vitamin D metabolites in the maternal and cord blood.

Blood samples	Indicator	Median	1st quartile	3rd quartile	*p*-value
Cord	Ferritin* [μg/l]	115.7	74.6	180.8	<0.001
Maternal		34.0	16.0	57.9	
Cord	Vitamin B12 [pg/ml]	301.0	207.0	440.0	0.279
Maternal		301.0	228.0	390.0	
Cord	Folic acid [ng/ml]	25.8	21.2	29.9	<0.001
Maternal		17.1	10.4	23.2	
Cord	25(OH)D3** [ng/ml]	23.3	19.7	29.4	<0.001
Maternal		44.7	35.6	52.3	
Cord	25(OH)D2** [ng/ml]	0.3	0.2	0.4	<0.001
Maternal		0.5	0.3	0.7	
Cord	24,25(OH)2D3** [ng/ml]	1.9	1.3	2.6	<0.001
Maternal		3.9	2.8	5.2	
Cord	3-epi-25(OH)D3** [ng/ml]	3.3	2.0	4.3	0.062
Maternal		3.4	2.3	4.3	
Cord	Total 25(OH)D** [ng/ml]	23.5	19.9	29.6	<0.001
Maternal		45.2	36.0	52.8	

*3 maternal and 6 neonatal cord blood samples were excluded due to increased ferritin levels associated with inflammation (*n* = 1) and preeclampsia (*n* = 2).

**3 maternal blood samples were excluded due to insufficient quantity or quality.

We noted no significant differences between maternal serum ferritin, vitamin B12 and vitamin D metabolites between women who did and did not supplement those micronutrients besides higher 24,25(OH)2D3 in the women who had supplemented vitamin D before pregnancy (Supplementary material 2). We investigated if the doses of supplements used both before and during pregnancy correlated with the results obtained from maternal samples. The only statistically significant association was demonstrated between the dose of vitamin D supplemented before pregnancy and the concentration of maternal 25(OH)D2 (*r* = 0.508, *p* = 0.006) (Supplementary material 3). We observed no significant differences in the concentrations of vitamin D metabolites in the maternal blood samples depending on the calendar season (*p* > 0.05).

### 3.4 Concentrations of the studied nutrients or their indicators in the umbilical cord blood

Concentrations of ferritin, vitamin B12, folic acid and vitamin D assessed in umbilical cord blood samples are presented in Table 3. The total 25(OH)D concentrations in the umbilical cord blood of <30 ng/mL were found in 77.5% of newborns (*n* = 79/102), and of <20 ng/mL in 25.5% (*n* = 26/102). For the remaining parameters, we did not identify reliable normal ranges for cord blood samples. However, in 19.6% (*n* = 20/102) of newborns, the cord blood vitamin B12 level was below the normal range and in 30% (*n* = 6/20) of those cases, a coexisting deficiency was noted in the mother. Only in 3.1% (*n* = 3/96) of cord blood samples, the ferritin level was below 30 μg/L and all mothers of those newborns also had low ferritin. The level of folic acid was not below the normal range proposed by the manufacturer in any of the cord blood samples.

### 3.5 Relations between the concentrations of the studied nutrients or their indicators in the maternal and umbilical cord blood

Relations between the concentrations of the selected nutrients or their indicators in cord and maternal blood samples were evaluated and significant differences were observed. The concentrations of ferritin and folic acid were significantly higher in cord blood samples than in maternal blood samples, whereas the concentrations of vitamin D metabolites were lower in cord blood samples in comparison with maternal samples except 3-epi-25(OH)D3 for which no significant differences were found. We determined no significant differences in the concentrations of vitamin B12 between maternal and cord blood samples (see details in Table 3). 3-epi-25(OH)D3 was detected in all cord blood samples and constituted the average of 8.1 and 14.5% of the total 25(OH)D concentration in the maternal and cord blood, respectively.

Correlations between the concentrations of selected indicators in the maternal and cord blood were assessed and significant weak to moderate positive correlations were found (Table 4).

**Table 4:** Correlations between selected micronutrient status indicators between the maternal and cord blood.

Indicator	Number of mother-newborn pairs	Spearman’s coefficient	*p*-value
Ferritin*	96	0.245	0.016
Vitamin B12	102	0.473	<0.001
Folic acid	102	0.279	0.004
25(OH)D3**	96	0.520	<0.001
25(OH)D2**	96	0.557	<0.001
24,25(OH)2D3**	96	0.590	<0.001
3-epi-25(OH)D3**	96	0.461	<0.001
Total 25(OH)D**	96	0.522	<0.001

*3 maternal and 6 neonatal cord blood samples were excluded due to increased ferritin levels associated with inflammation (*n* = 1) and preeclampsia (*n* = 2).

**3 maternal blood samples were excluded due to insufficient quantity or quality.

**Figure 2:**
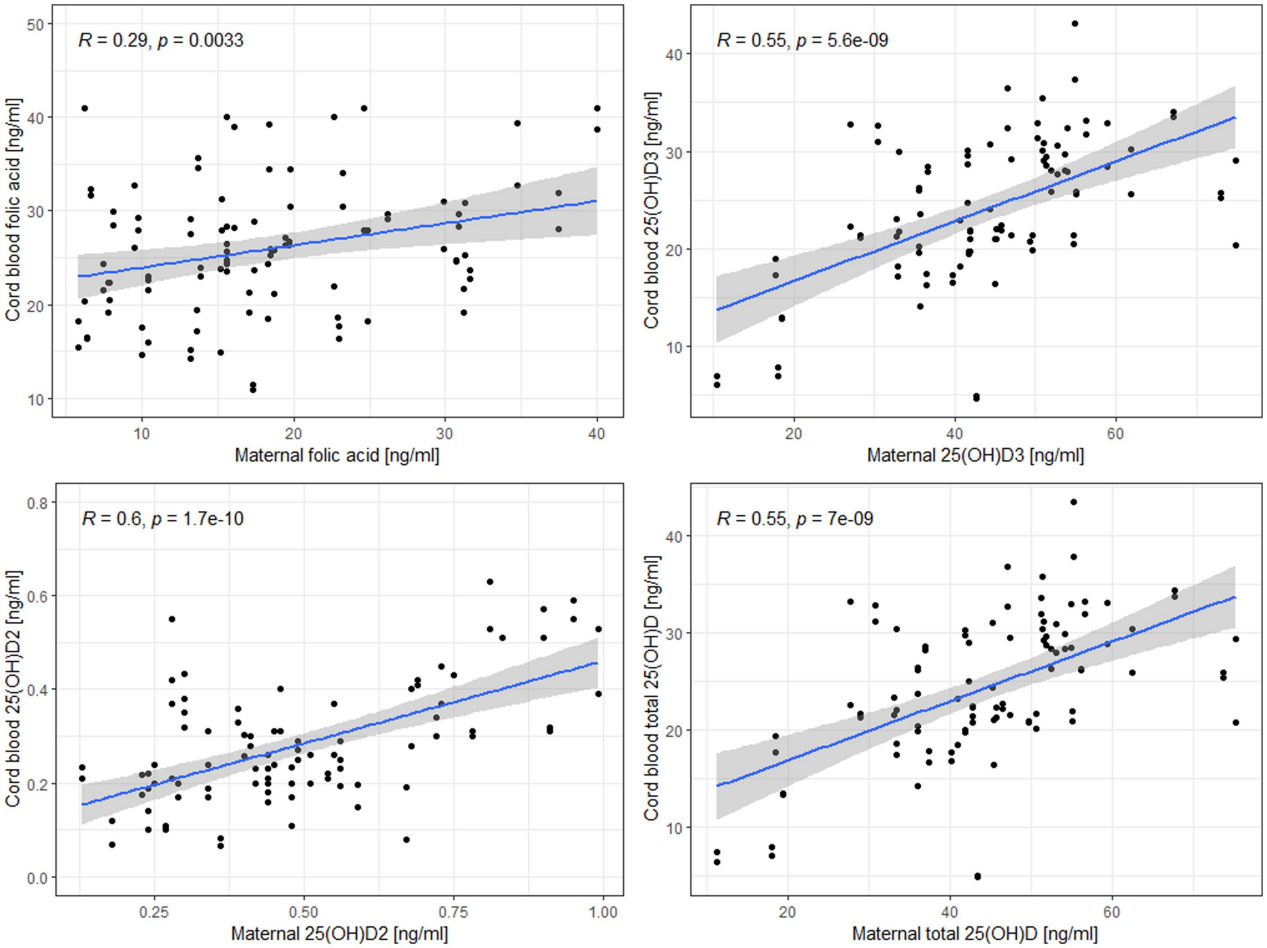
Linear regression models for the relationship between maternal and cord blood levels of folic acid, 25(OH)D2, 25(OH)D3, and total 25(OH)D.

Additionally, to assess the relationship between maternal and neonatal concentrations of the tested micronutrient supply indicators, a linear regression model was used with variables that presented a normal distribution after the removal of outliers: 25(OH)D2, 25(OH)D3, total 25(OH)D and folic acid (Figure 2). The fit of the model for folic acid was poor, i.e., it explained 8.3% of the variance. Models for vitamin D metabolites were better fitted and explained from 25.2 to 31.0% of the variance. An increase in maternal folic acid by 1 ug/l resulted in an increase in cord blood folic acid concentration by 0.24 ug/l. Similarly, an increase in maternal 25(OH)D3, 25(OH)D2 and total 25(OH)D resulted in an increase in cord blood 25(OH)D3, 25(OH)D2 and total 25(OH)D by 0.31, 0.39, 0.28 ng/mL, respectively (Supplementary material 4).

### 3.6 Relations between the assessed values of the studied nutrients or their indicators in the umbilical cord blood and maternal supplementation

We tested if there were differences in cord blood micronutrient status indicators between the newborns of women who had supplemented or had not supplemented a particular nutrient and found that pregestational folic acid and vitamin D supplementation was associated with higher cord blood levels of folic acid and 3-epi-25(OH)D3. Moreover, 25(OH)D3 and total 25(OH)D concentrations were statistically higher in newborns whose mothers had supplemented vitamin D during pregnancy (Table 5).

**Table 5:** The comparison of cord blood micronutrient supply indicators depending on the supplementation.

Supplementation	Cord blood indicators	Median	1st quartile	3rd quartile	*p*-value
Iron intragestationally YES *n* = 28 NO *n* = 9	Ferritin [μg/l]	114.0 128.0	77.0 83.2	182.0 214	0.592
Vitamin B12 intragestationally YES *n* = 19 NO *n* = 18	Vitamin B12 [pg/ml]	282.0 318.0	209.0 213.0	444.0 530.0	0.459
Folic acid pregestationally YES *n* = 19 NO *n* = 16	Folic acid [ng/ml]	27.7 21.4	22.3 17.1	29.9 24.6	0.001
Folic acid intragestationally YES *n* = 37 NO *n* = 0	Folic acid [ng/ml]	25.8	21.2	29.9	1.000
Vitamin D pregestationally YES *n* = 15 NO *n* = 20	25(OH)D3 [ng/ml]	26.8 21.1	20.8 17.3	30.1 26.1	0.056
	25(OH)D2 [ng/ml]	0.2 0.3	0.2 0.2	0.4 0.4	0.463
	24,25(OH)2D3 [ng/ml]	2.2 1.5	1.5 1.0	2.9 2.2	0.065
	3-epi-25(OH)D3 [ng/ml]	4.3 2.5	3.2 1.7	5.3 3.7	<0.001
	Total 25(OH)D [ng/ml]	27.0 21.3	20.9 17.7	30.3 26.3	0.056
Vitamin D intragestationally YES *n* = 35 NO *n* = 2	25(OH)D3 [ng/ml]	23.5 16.9	19.7 16.0	30.1 18.0	0.027
	25(OH)D2 [ng/ml]	0.3 0.2	0.2 0.2	0.4 0.3	0.558
	24,25(OH)2D3 [ng/ml]	1.9 1.6	1.2 1.0	2.6 2.4	0.641
	3-epi-25(OH)D3 [ng/ml]	3.4 2.7	2.0 1.6	4.5 3.8	0.430
	Total 25(OH)D [ng/ml]	23.8 17.2	19.9 16.2	30.3 18.3	0.025

The supplemented dosage and micronutrients were assessed for correlation with cord blood indicators and positive correlations were determined for vitamin B12, folic acid and 25(OH)D2 (Table 6).

**Table 6:** Correlations between the declared supplement doses of micronutrients and their supply indicators in the cord blood.

Supplementation	Cord blood indicators	Number of mother-newborn pairs*	Spearman’s coefficient	*p*-value
Iron intragestationally	Ferritin	56	−0.035	0.798
Vitamin B12 intragestationally	Vitamin B12	38	0.500	0.001
Folic acid pregestationally	Folic acid	38	0.380	0.001
Folic acid intragestationally	Folic acid	74	0.114	0.334
Vitamin D pregestationally	25(OH)D3	30	0.196	0.300
	25(OH)D2	30	0.381	0.038
	24,25(OH)2D3	30	0.149	0.431
	3-epi-25(OH)D3	30	0.039	0.816
	Total 25(OH)D	30	0.208	0.270
Vitamin D intragestationally	25(OH)D3	70	0.187	0.122
	25(OH)D2	70	0.273	0.022
	24,25(OH)2D3	70	−0.105	0.385
	3-epi-25(OH)D3	70	0.198	0.101
	Total 25(OH)D	70	0.195	0.105

*Samples from the newborns of women who had not supplemented a given micronutrient were not comprised in the calculations.

## 4 Discussion

Based on the above-presented results, we were able to observe that supplementation used by women in twin pregnancies, both before and during gestation, affected the composition of the umbilical cord blood, and that a significant correlation occurred between the concentrations of ferritin, vitamin B12, folic acid and vitamin D metabolites in the maternal and cord blood. However, deficiencies and non-compliance with supplementation recommendations were still a vivid problem. Our research group published a systematic review on micronutrients in multiple pregnancies before. Following the analysis of 12 articles and 1 series of publications, we concluded that women with multiple pregnancies might be at risk for vitamin D and iron deficiencies. As for other micronutrients, the data were scarce (6). Since then, several studies on this topic have been published. Le et al. found higher levels of 25(OH)D and vitamin D-binding protein in twin compared to singleton gestations and suggested that the assessment of vitamin D status in twin pregnancies should be treated with caution (25). Li et al. reported the deficiency or insufficiency of vitamin D (25(OH)D < 30 ng/mL) in over 40% of patients with dichorionic diamniotic pregnancies, and in as many as 99.3% of newborns, which was significantly higher than in our study (26). Conversely, Dera-Szymanowska et al., who compared the concentration of iron and calcium between singleton and twin pregnancies, found no differences between the groups in either maternal or cord blood samples (27).

Nevertheless, basing on our results, micronutrient undersupply seems a common problem in women with twin pregnancies in the perinatal period. Despite the widespread vitamin D supplementation and the prevalent use of both iron and vitamin B12, deficiencies were frequently demonstrated in the study group. No similar problems were found regarding folic acid. However, almost half of the participating women had not used folic acid in the preconception period. The results were unsatisfactory and only slightly higher than those presented by Wierzejska et al., who reported that only 42% of the respondents had supplemented folic acid pregestationally (28). In our group, the percentage of folic acid supplementation during pregnancy was higher – the substance was used by all the examined patients, compared to 83% reported by Wierzejska et al. (28) However, we found that, despite over a quarter of our study group were women who conceived by assisted reproductive techniques, and whose pregnancy was planned and monitored, 40.5% of the women did not use an adequate dose of folic acid.

Jankowska et al. who conducted a large study on the population of Polish pregnant women observed a similarly high intragestational supplementation of folic acid compared to our group (99.8% vs. 100%), while iron and vitamin B12 supplementation were less common than (23.6% vs. 75.7 and 38.5% vs. 51.4%, respectively). This may result not only from twin pregnancy itself, but also from the fact that Jankowska et al. recruited patients during the first half of pregnancy, while we examined our patients perinatally, and treatment could already be initiated due to the diagnosis of anemia (29). Regarding vitamin D supplementation, a similar frequency of vitamin D supplementation during pregnancy was described (up to 90%) in the study by Wierzejska et al. in the population of Polish women pregnant with singletons. However, the authors noted that a very low percentage of women met the recommendations for the daily consumption of vitamin D from the diet, and most of them used complex vitamin supplements, which often failed to contain the adequate doses of vitamin D (21, 30, 31).

Since our study was observational, the data regarding supplementation were based only on the patients’ declarations. We found no differences in maternal micronutrient supply indicators between groups supplementing or not a particular micronutrient, and maternal 25(OH)D2 concentration and vitamin D supplementation before pregnancy were the only significantly correlated parameters. This is particularly surprising because there are no supplements containing ergocalciferol available in Poland, and according to the literature, an inverse relationship was noted, i.e., a decrease in 25(OH)D2 after cholecalciferol supplementation (32). In our opinion, the lack of significant correlations regarding iron and vitamin B12 might result from the fact that, according to the Polish recommendations, they are administered mainly after establishing the diagnosis of deficiency anemia (31).

We found that the declared pregestational supplementation of folic acid and both pre-and intragestational supplementation of vitamin D affected the concentrations of their metabolites in the cord blood and that the dosage of vitamin B12 was positively correlated with its cord blood levels. In our opinion, the association of pregestational supplementation and cord blood results was most likely due to the fact that women who had taken supplements before pregnancy had used them for a long time, which corresponded to the actual better supply of the mother’s body. Nevertheless, it needs to be mentioned that this did not find expression in the comparison of folic acid concentrations between groups supplementing it before pregnancy and not. Nevertheless, authors believe, that its metabolism is complex and the concentration of one metabolite may not be sufficient to fully evaluate the supply. Our results correspond with the studies published so far, mostly conducted in the population of singleton pregnancies. A randomized controlled study by Li et al., who administered a preparation with 2 μg of cobalamin to pregnant patients, showed that the newborns of mothers who received vitamin B complex supplements had higher cord vitamin B12 concentrations (33). Maulik et al. found that prenatal supplementation increased the concentrations of 5-methyltetrahydrofolate, i.e., the most prevalent folate, in both maternal and cord blood samples (34). Similarly, McNulty et al. conducted a study concerning continuous folic acid supplementation throughout the pregnancy and concluded that 2nd and 3rd trimester supplementation might result in an increased maternal and cord blood folate status (35). In our study, the pregestational supplementation of vitamin D was associated with higher levels of 3-epi-25(OH)D3. Such an association had also been already confirmed by researchers, but the role of epimers has not been elucidated yet (36). Schulze et al. tested the effect of multiple micronutrient supplementation and observed that it might result in increased cord ferritin and 25(OH)D levels, while maternal levels of cobalamin, folate and 25(OH)D were correlated with cord blood levels (37).

In the present study, we found a significant correlation between the maternal and twin cord blood levels of all studied indicators. Therefore, we may hypothesize that the better the maternal supply, the better the supply of the newborn. However, ferritin levels seem more difficult to interpret since, in the light of recent research, its role turns out to be more complex. Previously, Delaney et al. evaluated iron status indicators in 234 samples of the cord blood, 46.2% of which were obtained from multiple pregnancies. They observed a significant inverse relationship between cord ferritin and hemoglobin concentrations that was not referable to inflammatory markers (38). Such an association was linked to the preferential use of iron in erythropoiesis, which, however, explains the phenomenon partially (38, 39). However, the cord blood level of ferritin in the subgroup of multiple pregnancies in this study was similar to the one that we reported (103.3 vs. 115.7 μg/L) (28). As mentioned, the interpretation of the relationship between iron supplementation and the results in the maternal and cord blood is further complicated by the fact that iron is not a permanent component of pregnancy supplementation, and it is often taken by patients with already diagnosed deficiencies.

Previously published data on the relations between folic acid concentrations in the maternal and umbilical cord blood were ambiguous. Soliburska et al. conducted a study in a group of singleton pregnancies and, similarly to us, reported a significant positive correlation between maternal and cord blood folic acid concentrations (40). Comparably to Du et al. in singleton pregnancies, we found higher folate concentrations in the cord blood in our twin cohort (41). However, Plumptre et al. found cord serum folate concentrations to be 64% higher than those of the maternal serum, while Maulik et al. reported higher 5-methyltetrahydrofolate, 5,10-methenyl-tetrahydrofolate, tetrahydrofolate and lower folic acid concentrations in the cord blood compared to maternal samples (34, 42).

Du et al., Surekha et al. and Obeid et al. demonstrated higher concentrations of vitamin B12 in the umbilical cord blood than in the maternal blood (41, 43, 44). In our study, no differences were noted in vitamin B12 levels between the maternal and cord blood, but, like the above mentioned authors, we found a significant correlation of concentrations between the maternal and cord blood. Interestingly, Du et al. observed that the levels of folate and vitamin B12 increased nonlinearly in the umbilical cord with their increase in maternal samples which presented as an inverted U-shaped curve (41).

We reported higher concentrations of most vitamin D metabolites in the maternal samples compared to the umbilical cord blood. A similar study was conducted by Wierzejska et al. in a cohort of Polish women with singleton pregnancies. The authors found a lower concentration of 25(OH)D in the maternal serum compared to the cord blood and a very high incidence of maternal vitamin D deficiency or insufficiency, totaling 89%, whereas in our cohort it accounted for only 12.5% (45). Conversely, the cord blood 25(OH) level of <20 ng/mL was found in 28.7% of cases, which was a similar result to ours (25.5%) (45). However, the main difference between the studies is the laboratory method used. In the study by Wierzejska et al., immunological methods, while in our study, liquid chromatography with tandem mass spectrometry (LC–MS/MS) were applied. In a previously published study conducted in singleton and twin pregnancies, we found that the use of immunological methods resulted in lower 25(OH)D measurements than LC–MS/MS and they could not be used interchangeably (20) Additionally, Lu et al. described the phenomenon of the overestimation of cord blood 25(OH)D levels using immunoassays, so the LC–MS/MS method seems to be more reliable (46). Therefore, our results are more consistent with those published by Mao et al. who used a similar methodology (47). Our results are also different to those by Goswami et al., whodemonstrated a high percentage of deficiencies and lower maternal and neonatal concentrations of this metabolite in twin pregnancies. Nevertheless, those authors also used immunoassays and studied a completely different population from ours, i.e., one originating from India, where vitamin D supplementation is not routinely used in pregnancy (8).

The advantages of our study include a considerable group of patients with twin pregnancies compared to other available studies. The obtained results were correlated with numerous collected data, such as chorionicity or the information on supplementation before and during pregnancy. In our study, we also used the gold diagnostic standard for vitamin D, i.e., LC–MS/MS, which allowed for a reliable assessment of the metabolite profile. The determination of vitamin D status using LC–MS/MS offers the possibility of the isolation of other metabolites, e.g., 3-epi-25(OH)D3. It is known for its lower affinity for the vitamin D receptor than 25(OH)D. Therefore, it is believed that cross-reactivity may occur in immunological methods, which may be conducive to the underestimation of vitamin D deficiency (48–51). In our study, the concentrations of the above mentioned epimer constituted the average of 8.1 and 14.5% of the total 25(OH)D concentrations in the maternal and cord blood, respectively, which may also contribute to clinical implications and misclassification. The limitations of the study include missing data, particularly regarding supplementation, and the fact that the recruitment was conducted at a single center. Moreover, we did not assess all parameters of iron, folic acid and vitamin B12 metabolism, which may narrow the perspective. Another disadvantage was the observational nature, a variety of supplementation used by the patients without detailed information, for example concerning the type of iron supplement used. Lastly, the effects of micronutrient supplementation on health outcomes may become evident only with longitudinal follow-up of pregnant women and the neonates.

## 5 Conclusion

Despite the widespread use of supplementation, the problem of deficiencies in the population of women with twin pregnancies in terms of iron, vitamin B12 and vitamin D is still vivid. A large percentage of patients still do not use the recommended supplementation before and during pregnancy, and this is especially important considering that both pregestational and intragestational supplementation affects the cord blood composition of twins. However, an optimal micronutrient supply and supplementation in twin pregnancies that results in the best maternal and neonatal outcomes is yet to be determined.

## Data Availability

The raw data supporting the conclusions of this article will be made available by the authors, without undue reservation.
