# Supplementary material for: Assessment of the maternal key micronutrient supply and its correlation with cord blood parameters in twin pregnancies
Source: Front Public Health. 2025 Feb 25;13:1487730. doi: 10.3389/fpubh.2025.1487730 (PMC11895532; doi:10.3389/fpubh.2025.1487730)
Supplement: Supplementary file 1 [file Table_1.DOCX]

Supplementary Material 1. Comparison of monochorionic and dichorionic pregnancies.

|  | Monochorionic pregnancies  (n=19 pregnant,  38 neonates) | Dichorionic  pregnancies  (n=32 pregnant, 64 neonates) | p-value |
| --- | --- | --- | --- |
| Pregnant women | | | |
| Maternal age [years] | 31.0  (30.0; 36.0) | 33.5  (29.0; 35.0) | 0.914 |
| Parity  ⋅Primiparous  ⋅Multiparous | 42.1 (8)  57.9 (11) | 46.9 (15)  53.1 (17) | 0.779 |
| Conception*  ⋅Spontaneous  ⋅ART | 78.9 (15)  21.1 (4) | 68.7 (22)  31.3 (10) | 0.527 |
| Pregestational BMI [kg/m^2^] | 22.1  (20.4; 24.2) | 24.7  (22.0; 27.8) | 0.005 |
| Pregnancy complications*  ⋅GDM  ⋅HDP  ⋅PB | 26.3 (5)  10.5 (2)  84.2 (16) | 0 (0)  6.3 (2)  46.9 (15) | 0.005  0.623  0.008 |
| Gestational weight gain [kg] | 17  (11; 22) | 16  (10; 21) | 0.519 |
| Gestational age at birth [weeks+days] | 36+1  (34+5; 36+6) | 37+0  (35+4; 37+1) | 0.004 |
| Neonates | | | |
| Birth weight [grams] | 2340  (1650; 2640) | 2510  (2190; 2750) | 0.038 |
| Initial Apgar score*  [points]  8-10  4-7  0-3 | 94.7 (36)  5.3 (2)  0.0 (0) | 93.8 (60)  6.2 (4)  0.0 (0) | 1.000 |

*- % (number)

ART – Assisted Reproductive Technology; BMI – Body Mass Index; GDM – Gestational Diabetes Mellitus; HDP – Hypertensive Disorders of Pregnancy; PB – Preterm Birth

Supplementary Material 2. Comparison of maternal micronutrient supply indicators depending on the supplementation.

| Supplementation | Maternal supply indicator | Median | 1^st^ quartile | 3^rd^ quartile | p-value |
| --- | --- | --- | --- | --- | --- |
| Iron during pregnancy  YES n=28  NO n=9 | Ferritin  [µg/l] | 26.4  30.8 | 15.7  11.2 | 50.2  56.2 | 0.350 |
| Vitamin B12 during pregnancy  YES n=19  NO n=18 | Vitamin B12  [pg/ml] | 301.0  289.0 | 228.0  185.0 | 390.0  420.0 | 0.685 |
| Folic acid prepregnancy  YES n=19  NO n=16 | Folic acid  [ng/ml] | 15.6  14.8 | 10.4  10.2 | 22.7  23.0 | 0.608 |
| Folic acid during pregnancy  YES=37  NO=0 | Folic acid  [ng/ml] | 17.1 | 10.4 | 23.2 | 1.000 |
| Vitamin D prepregnancy  YES=15  NO=20 | 25(OH)D3  [ng/ml] | 43.0  43.9 | 35.5  35.5 | 54.0  50.3 | 0.574 |
|  | 25(OH)D2  [ng/ml] | 0.5  0.5 | 0.3  0.4 | 0.6  0.7 | 0.347 |
|  | 24,25(OH)2D3  [ng/ml] | 4.6  3.6 | 3.1  2.0 | 5.4  4.5 | 0.023 |
|  | 3-epi-25(OH)D3  [ng/ml] | 4.0  3.4 | 2.5  2.3 | 4.4  4.3 | 0.320 |
|  | Total 25(OH)D  [ng/ml] | 43.7  44.5 | 36.0  36.0 | 54.9  51.1 | 0.538 |
| Vitamin D during pregnancy  YES=35  NO=2 | 25(OH)D3  [ng/ml] | 43.6  39.7 | 35.5  39.7 | 52.7  39.7 | 0.492 |
|  | 25(OH)D2  [ng/ml] | 0.5  0.5 | 0.3  0.5 | 0.7  0.5 | 0.902 |
|  | 24,25(OH)2D3  [ng/ml] | 3.8  3.4 | 2.3  3.4 | 5.0  3.4 | 0.585 |
|  | 3-epi-25(OH)D3  [ng/ml] | 3.5  1.9 | 2.3  1.9 | 4.3  1.9 | 0.125 |
|  | Total 25(OH)D  [ng/ml] | 44.3  40.2 | 36.0  40.2 | 53.1  40.2 | 0.492 |

Supplementary Material 3. Correlation between the dosage of supplements before and during pregnancy with the maternal micronutrient supply indicators.

| Supplementation | Maternal micronutrient supply indicators | Spearman coefficient | p-value |
| --- | --- | --- | --- |
| Iron during pregnancy | Ferritin | -0.046 | 0.746 |
| Vitamin B12 during pregnancy | Vitamin B12 | 0.146 | 0.382 |
| Folic acid prepregnancy | Folic acid | -0.235 | 0.156 |
| Folic acid during pregnancy | Folic acid | -0.050 | 0.671 |
| Vitamin D prepregnancy | 25(OH)D3 | 0.213 | 0.277 |
|  | 25(OH)D2 | 0.508 | 0.006 |
|  | 24,25(OH)2D3 | 0.036 | 0.855 |
|  | 3-epi-25(OH)D3 | 0.177 | 0.368 |
|  | Total 25(OH)D | 0.213 | 0.277 |
| Vitamin D during pregnancy | 25(OH)D3 | 0.190 | 0.121 |
|  | 25(OH)D2 | 0.068 | 0.022 |
|  | 24,25(OH)2D3 | 0.044 | 0.723 |
|  | 3-epi-25(OH)D3 | -0.008 | 0.948 |
|  | Total 25(OH)D | 0.200 | 0.102 |

Supplementary Material 4. Linear regression models for the relationship between maternal and cord blood levels of folic acid, 25(OH)D2, 25(OH)D3, total 25(OH)D.

| **Term** | **Estimate** | **SE** | **p-value** | **R2 coefficient** |
| --- | --- | --- | --- | --- |
| (Intercept) | 21.60 | 1.59 | <0.001 | 0.083 |
| Maternal folic acid level [ug/l] | 0.24 | 0.08 | 0.003 |  |
| (Intercept) | 10.56 | 2.19 | <0.001 | 0.305 |
| Maternal 25(OH)D3 level [ng/ml] | 0.31 | 0.05 | <0.001 |  |
| (Intercept) | 0.10 | 0.03 | <0.001 | 0.310 |
| Maternal 25(OH)D2 level [ng/ml] | 0.39 | 0.06 | <0.001 |  |
| (Intercept) | 12.3 | 2.32 | <0.001 | 0.25 |
| Maternal total 25(OH)D level [ng/ml] | 0.28 | 0.05 | <0.001 |  |

SE – standard error
